# A reversal of age-dependent proliferative capacity of endothelial progenitor cells from different species origin in in vitro condition

**DOI:** 10.15171/jcvtr.2016.22

**Published:** 2016-09-26

**Authors:** Mehdi Hassanpour, Omid Cheraghi, Vahid Siavashi, Reza Rahbarghazi, Mohammad Nouri

**Affiliations:** ^1^Department of Clinical Biochemistry and Laboratory Medicine, Tabriz University of Medical Sciences, Tabriz, Iran; ^2^Department of Biology, Faculty of Natural Sciences, University of Tabriz, Tabriz, Iran; ^3^Stem Cell Research Center, Tabriz University of Medical Sciences, Tabriz, Iran; ^4^Department of Clinical Pathology, Faculty of Veterinary Medicine, University of Tehran, Tehran, Iran

**Keywords:** Endothelial Progenitor Cells, Human, Mouse, Dog, Aging

## Abstract

***Introduction: ***A large number of cardiovascular disorders and abnormalities, notably accelerated vascular deficiencies could be related to aging changes and increased length of life. During the past decades, the discovery of different stem cells facilitates ongoing attempts for attenuating many disorders, especially in vascular beds. Endothelial progenitor cells (EPCs) are a subtype of stem cells that have potent capacity to differentiate into mature endothelial cells (ECs). However, some documented studies reported an age-related decline in proliferation and function of many stem cells. There is no data on aging effect upon proliferation and morphological feature of EPCs.

***Methods: ***To show aging effect on EPCs proliferation and multipotentiality, bone marrow samples were provided from old and young cases in three different species; human, mouse and dog. After 7 days of culture, the cell morphology and clonogenic capacity were evaluated. We also calculated the mean number of colonies both in bone marrow samples from old and young subjects. To confirm the cell phenotype, isolated cells were immune-phenotyped by a panel of antibodies against Tie-2, CD133 and CD309 markers.

***Results:*** Our results showed that EPCs exhibited prominent spindle form in all bone marrow samples from young cases while the cell shape became more round by aging. Notably, the number of colonies was reduced in aged samples as compared to parallel young subject samples (*P* < 0.05). We also detected that the expression of endothelial related markers diminished by aging.

***Conclusion: ***The results of this study suggest that the age-related vascular abnormalities could be presumably related to the decline in stemness capacity of EPCs.

## Introduction

Cardio vascular disease, notably myocardial infarction, is the leading globally cause of death and accounting for approximately 17.3 million annual mortalities.^1^ Based on given statistics, post-ischemic complications happen due to vascular abnormalities. In this condition, regeneration capacity by the recovery performance of coronary artery and lateral branches decreased strongly with increased length of life. In addition to current conventional therapies for amelioration of cardiac insufficiency, an emerging field of stem cells therapy sheds promising lights to minimize abnormal functionalities.^2,3^

In this regard, bone marrow medulla contains different subgroups of progenitor cells with potent ability to give rise to various mature end-stage cells.^4^ Notably, Asahara et al initially identified endothelial progenitor cells (EPCs) which further trans-differentiate into mature endothelial cells (ECs).^5^ As commonly conceived, no unique cell-surface antigens have been described yet.^6^ A multiple of cell-surface panel, including CD133, Tie-2, vascular endothelial growth factor-2 (VEGFR-2; also known as KDR1 or CD309), CD34, Von Willebrand factor has been however used extensively to characterize EPCs.^7^ In response to the chemotactic gradient, these cells easily recruit to ischemic regions and result in *de novo* formation of vascular beds.^8^

At the moment, some difficulties such as isolation of insufficient cell number and slow expansion of EPCs in in vitro condition have contributed to incomplete understanding of the cell biology and conception of angiogenesis-related mechanism especially regarding to EPCs.^9^ Therefore, many efforts have been undertaken to improve cell harvest procedure and medium favoring EPCs cultivation and expansion in in vitro condition.^10,11^ However, recent experiments unveiled the effect of many factors such as the time of cultivation, media source, separation method, different growth factor supplementation and culture surface could alter the pattern of EPC-related cell biomarkers and kinetics.^12^

Like many surrounding agents who affecting the hemostatic and regenerative capacity of normal cells - in particular stem cells - residing in numerous tissues, aging is a prominent and substantial issue.^13^ Not only adult stem cells, but also their committed progenies self-renewability capacity is completely influenced by age-related changes.^13^ It is well-established that the active hemostasis of proteomic and genomic prominent machineries would be abolished by age.^13,14^ For instance, age-related DNA modifications happen by phosphorylation of H2AX, acetylation of H3K56 and methylation of H3K79.^15,16^

Additionally, based on validated data, the number of circulating EPCs reduced reversibly with aging particularly in patients with coronary artery disease.^17^ For example, Vasa et al reported age-associated decline in circulating number of CD34/CD309-positive cells in a mixed group of aged healthy individuals and even patients with a recent myocardial infarction.^18^

To better understand the effect of age-related changes on EPCs proliferation capacity, different EPCs from three distinct species, namely human, dog and mouse, were provided from aged and young subjects.

## Materials and Methods

### EPCs isolation and expansion procedure from different species

### Human samples

Bone marrow aspirates which presented to a clinical laboratory of Shahid Ghazi hospital and Children hospital, affiliated hospitals to Tabriz University of Medical Sciences, were used in the current study. After obtaining written permission form, the remaining samples from healthy volunteers ranging from 3 to 70 years old subjected to EPC isolation. In brief, a volume of 3 ml bone marrow aspirate containing heparin, diluted with phosphate buffered saline solution (PBS) at the ratio of 1:3, overlaid on Ficoll-Hypaque (Sigma) and then centrifuged at 400 g for 20 minutes at 4°C. Thereafter, mononuclear cells at Ficoll and plasma interphase were collected, washed twice with PBS. An initial density of 1 × 10^5^ cells per cm^2^ in M199 culture medium (Gibco) containing 2% fetal bovine serum (FBS; Gibco) and supplemented with EGM-2 BulletKit (Cat No: C-39211; Promocell) seeded on pre-coated fibronectin (1 µg/mL; Promocell) and maintained in humidified atmosphere with 7% CO_2_ at 37°C. The exhausted medium was further replenished every four days.

### Murine samples

To compare the proliferation behavior of expanded mEPCs form aged (18 month-old) and immature (2 week-old) mice with human counterpart, mononuclear cells from both neonatal and mice at 2 months of age were harvested. After euthanasia, both femurs of each mouse were taken by muscular excision. Thereafter, epiphyses were ruptured, and a 20-gauge needle syringe containing the culture medium M199 was inserted in one of the bone extremities while the opposite side was located in a 10 cm culture dish. Medullary contents rigidly flashed out by repeated back-and-forth movements. After that, M199 medium containing bone marrow cells was overlaid on equal volume of Ficoll Hypaque, centrifuged at 400 g for 20 minutes. Further, mononuclear cells were isolated from middle phase and washed with PBS and centrifuged at 400 g for 5 minutes. Finally, the isolated cells were cultured in conditions similar to those cells of human cells.

### Canine samples

In line with human and murine samples, cEPCs culture was also provided. In this study, the bone marrow samples from 6-month-old and dogs at the age of a 6-year-old were prepared and underwent to EPC protocol culture as above-mentioned.

### Morphological phenotype of cultured EPCs from different species sources

Seven days after cell seeding, we analyzed clonogenicity of cultivated cells under our conditions. The number of EPCs colonies was manually counted in five random high-power fields. We also monitored the morphological characteristics of cells, using by an inverted microscopy 7 days after initial plating.

### Flow cytometric immunophenotypic analysis of EPCs

For characterization of isolated cells, the surface markers of EPCs were analyzed by using flow cytometric assay. After day-7 of culture bone marrow cells in EPC-associated medium, cells were detached with 0.25% Trypsin-EDTA solution, neutralized by FBS, and washed twice with PBS. Thereafter, a panel of antibodies directed against PE mouse anti-human CD309 (BD Pharmingen), PE mouse anti-human CD31 (ebioscience), FITC mouse anti-human CD133 (Miltenyi BioTec) and Tie-2 (mouse anti-human Tie-2, Abcam) and goat anti-mouse IgG-Texas red (Abcam) were provided. Briefly, an approximate number of 5 × 10^5^ to 10^6^ cells blocked by 2% FBS solution for 15 minutes, and incubated with 100 µL PBS containing manufacturer’s recommended concentration for 30 minutes, washed twice with PBS and fixed in 4% paraformaldehyde (PFA). Ultimately, the percentage of EPC marker positive cells analyzed by BD FACSCalibur^™^ flow cytometer and final raw data processed via FlowJo software version 7.6.1.

### Statistical analysis

In this study, data are expressed as mean ± standard deviation (Mean ± SD) and analyzed by using the GraphPad InStat package version 2.02 (GraphPad Software Inc.). The data analyses between groups were also performed via the one-way analysis of variance (ANOVA) with Tukey post hoc test with a significant level at *P *< 0.05. In histograms, statistical difference between the groups presented by brackets with **P < 0.01 and ***P < 0.001.

## Results

### Morphological characteristics

**Figure 1:**
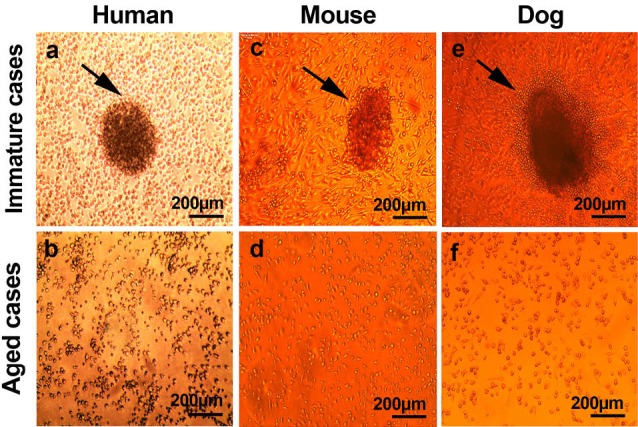
Light microscopic images of EPCs from three different species (a-f). As seen, EPCs tend to form colony forming unites in all immature cases 7 days after plating (a, c and e). No obvious clonogenic capacity was evident in bone marrow samples from aged subjects (b, d and f).

Seven-day post-seeding, fibronectin-coated plates induced a spindle- to round-like morphology in the majority of cultivated cells from immature cases; although some cobble stone-shaped cells were also evident (Figure 1a-f). We found that numerous round cell clusters appeared within seven days post-plating while spindle-shaped cells, which were detected adhered to the surface, started to sprout from the cluster core (Figure 1a, c and e). In contrary to cells isolated from immature cases, only round-shaped cells were seen in aged samples prepared from three different species (Figure 1b, d and F). Therefore, we concluded that cell shape; morphology and micro-aggregation capacity under in EPC specific medium could be altered with advancing of age.

### The clonogenic EPCs capacity decreased with aging

**Figure 2:**
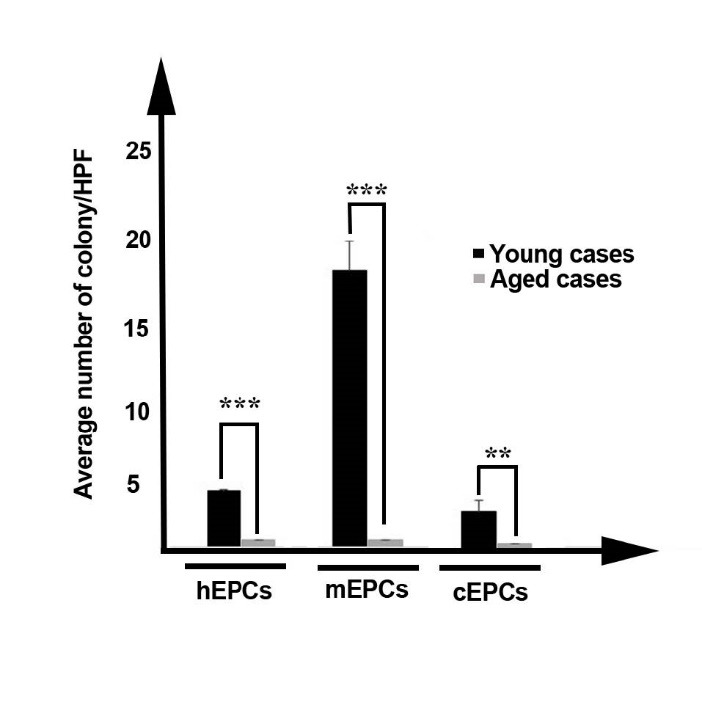
Representative average number of colonies per high power field. Morphological observation showed a significance difference in colony number and shape of colony between immature and aged groups. The mean number of colonies in aged groups was significantly more than aged groups. Differences between control and treated groups are significant at **P < 0.01, and ***P < 0.001.

Clonogenicity is indicative and reliable for identification different progenitor stem cells such as EPCs. Therefore, we assessed in vitro clonogenic capacity of EPCs in samples from aged and immature cases in three species. In accordance with our results, a significant decline in clonogenic potential of aged-related EPCs, by decreasing the mean number of EPCs colony forming unit, was evident as compared to immature counterparts (*P*_aged-hEPC versus neonatal hEPC_ and _aged-mEPC versus immature mEPC_ < 0.001; *P*_aged-cEPC versus immature cEPC_ < 0.01; Figure 2).

### Immunophenotypic characteristics of isolated EPCs revealed

**Figure 3:**
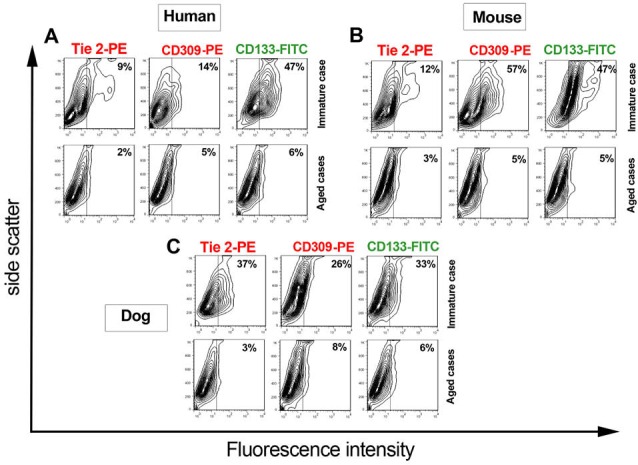
Flow cytometric characterization of human, murine and canine EPCs. Cells were incubated with a panel of antibodies including Tie2-PE, CD309-PE and CD133-FITC and analyzed with BD FACS flow cytometry system. The data showed an increase in the number of CD133, Tie-2 and CD309-2 positive cells by aging. The data are expressed as mean ± SD (n = 6).

By 7-day of culture of isolated cells, the identity of the cultured cells was investigated using flow cytometry immunophenotypic analysis. In human samples, our results showed that cultured immature hEPCs yielded a cell population with 47% CD133^+^, 14% CD309^+^ and 9% Tie-2^+^ values whereas the expressions of these markers in aged subjects were 6, 5 and 2% respectively (Figure 3a). The cells originated from immature murine samples represented 47 CD133^+^, 57 CD309^+^ and 12% Tie-2^+^ positive cells. Similar to results of aged human subjects, a same pattern of reactivity against CD133, CD309 and Tie-2, in order of 47, 57 and 12 percent, was achieved (Figure 3b). The comparison of obtained data from canine samples with human and murine counterparts also revealed that in dogs the percentage of EPC marker positive cells decreased with aging (Figure 3c). In detail, 26, 18 and 34% reduction in CD133, CD309 and Tie-2 positive cells were determined. Collectively, these outcomes confirmed that EPC-related antigens were clearly decreased in each three aged subject in compression with parallel immature counterparts.

## Discussion

Advancing age is associated with impaired function of different organs notably cardiovascular system. It is particular significance to preserve the structure and function of vascular bed during post-injury sequelae through a life-long period.^19^ According to literature, the onset of progressive senile changes is correlated with a declined rate of angiogenesis in old-age subjects.^20^ Additionally, an in vitro expansion of different stem cells from aged donors represented indicative features of chronological and replicative aging.^21^ Angiogenesis is de novo formation of capillaries from pre-existing capillary plexus.^22^ A plethora of findings revealed that EPCs, as EC progenitor, are an eligible source of a cellular pool to retain or return vessel regeneration during different injuries among the scientific community.^4^

To better understand whether advancing ages affect EC progenitor cells, EPCs growth characteristics. Therefore, we for the first time evaluated the effect of aged status on in vitro proliferation and expansion capacity and clonogenicity in three different species (i.e. human, dog and mouse). In current experiment, bone marrow samples were provided from both aged and immature animal cases and human subjects. Regarding the results of our study, a 7-day incubation of isolated bone marrow mononuclear cells from different non-aged species resulted in formation of primary out-growth EPC colonies under EPC induction medium while no evidence of colonies was detectable in samples of aged donors. In addition to morphological assessment, flow cytometric analysis did not reveal the existence of EPC-associated markers (i.e. Tie-2, CD309 and even CD133) in aged cases as compared to non-aged subjects. It was revealed that CD133 actively participated in self-renewability and colonosphere formation of stem cells driven by Src-family kinase, PI3K/Akt pathways.^23,24^ In line with our results, Povsic et al recently acclaimed that the absolute number of circulating CD34, CD133 and CD309-positive cells with decreased by aging.^25^ In addition, it has shown that tyrosine kinase activity and proliferative capacity of choroid plexus regarding Tie-2 and CD309 expression decreased with aging in response to angiogenic stimuli in a rat model.^26^ Up to present, some authorities declared an inevitable role of strain and genetic differences in the number of circulating EPCs.^27^ For instance, Shaked and colleagues proved an increased number of systemic EPC with age advancing in a murine model,^27^ but it remains to be elucidated that whether genetic specificity could result in high rate of circulating EPCs through bone marrow potent capacity to be produced and released or a dynamics of active angiogenesis-dependent signaling orchestrates continuous recall of EPC from bone marrow niche.^28^

In another experiment directed by Wang et al, it was established an increased number of resident CD133 positive cells in aged rat bone marrow medullary space while a prominent down-regulation in expression of CD26 coincided with a declined proteolytic enzymes activity resulted in cell mobilization to extra-medullary niche.^29^ With these descriptions, it is possible to infer that senesce could influence the function of mechanism governing angiogenesis in addition to EPCs growth characteristics, trans-differentiation and kinetics. In spite of these interpretations, it was previously aforementioned the age-associated impairments apparently have been occurred in EPC number and function due to a variety of environmental changes that thereby alter the generation and mobilization of EPC from the bone marrow, subsequent homing and function by an oriented modulation of intracellular signaling.^30^ On the other hand, the high level of angiogenic factors in plasma notably; VEGF could not enhance the expression of CD309 in aged non-healthy subjects.^28^ In our study, age inversely correlated with EPC growth characteristics in which aged subjects did not show EPC clusters as compared to young counter parts. Therefore, it is logical that a large number of subjects should be evaluated to confirm our results in the future analysis.

There are some limitations in analysis of EPC immunophenotyping in the current experiment. It is noteworthy to mention that no definite cells markers exist for the precise discrimination between EPCs and hematopoietic progenitor cells and given that many phenotypic traits could be expressed at same time in both lineages. Hence, we suggest targeting definite specific negative markers or using other sophisticated tools, such as proteomic and genomic approaches, for better identification of EPC dynamics in different milieus. Here, we did not purify the expanded EPCs after 7 days. It seems that cell enrichment or sorting techniques could be useful in the achievement of desired cell populations for accurate monitoring.

Assuming cell markers, we previously determined that early EPCs are negative for CD11b, CD14 and CD45 with low proliferative rate available after only 7 days of growth on fibronectin-coated dishes.^31^ Late EPCs are highly proliferative ECs emerging 14 to 21 days on fibronectin-coated dishes, acquiring more endothelial differentiation traits, such as the cobblestone morphology. Also, they are CD14, CD45 and CD115 negative. In addition to morphologic appearance and clonogenicity, these traits could be used for distinguishing from hematopoietic cells.^31^

Conclusively, we showed that cell proliferation of EPCs is affected by aging not only in human, but also in other species. Our data further support EPCs proliferative capacity reflects the patient’s potency to repair ongoing injuries and insults. Our experiment shed lights on previously published evidence that the reduced regenerative of aged EPCs is mechanistic explanation for insufficient neo-angiogenesis with biological aging. However, more experiments are needed to precisely elaborate the effects of aging on proliferative capacity of EPCs.

## Ethical approval

All procedures performed in studies involving human participants were identical to the ethical standards of the institutional and/or national research committee and with the 1964 Helsinki declaration. In addition, all animals were manipulated in accordance with the published guideline of the Care and Use of Laboratory Animals (NIH Publication No. 85-23, revised 1996) and approved by the Animal Care Committee of the Tabriz University of Medical Sciences.

## Competing interests

All authors declare no competing financial interests exist.
